# Impact of a Talent Development Environment on Sport Commitment: The Case of Chinese Athletes

**DOI:** 10.5114/jhk/208751

**Published:** 2025-09-23

**Authors:** Xiaolong Zhang, Xin Wei

**Affiliations:** 1 School of Physical Education, Hubei University of Technology, Wuhan, China.

**Keywords:** athlete retention, sub-elite and elite differentiation, Chinese sports system

## Abstract

The study aimed to examine the effect of various factors in the talent development environment (TDE) on the sport commitment of Chinese athletes and whether this effect differed across various sport levels. We surveyed 176 Chinese athletes using a questionnaire. Stepwise regression and five-fold cross-validation were used to identify key factors in the TDE that influenced sport commitment. Multiple linear regression was then used to examine differences across sport levels. Long-term development focus had a significant positive effect on the sport commitment of sub-elite and elite athletes (p < 0.01 and p < 0.05, respectively). The support network negatively affected sport commitment only in sub-elite athletes (p < 0.01), while long-term development fundamentals positively influenced sport commitment only in elite athletes (p < 0.05). Creating a TDE that aligns with the requirements of long-term development focus is beneficial for enhancing sport commitment. The application of support networks should be directed toward long-term success while avoiding excessive emphasis on short-term success. Although the stage-specific nature of athlete development and the unique characteristics of the China’s sports system may limit the immediate effect of long-term development fundamentals on sport commitment in the early stages of an athlete’s career, their role in preventing burnout underscores their continued importance. This study provides a theoretical foundation and practical guidance for enhancing sport commitment and offers a potential pathway to addressing premature retirement among Chinese athletes.

## Introduction

In China, premature retirement among athletes occurs frequently (Ling and Hong, 2014; Zhang, 2024). Many outstanding athletes choose to retire during their prime athletic years. This phenomenon not only leads to a significant waste of the country’s investment in competitive sports, but also affects the accumulation of high-level athletic talent and experience (Zhou and Chen, 2004). Focusing on sport commitment has been shown to effectively mitigate this phenomenon (Swan et al., 2022). Sport commitment, as a psychological state, represents an individual’s desire and determination to maintain participation in sports (Scanlan et al., 1993). Building on this, Carpenter et al. (1993) proposed that sport enjoyment, personal investment, and involvement opportunities have a positive effect on sport commitment, while social constraints have a negative effect. Subsequent research has introduced additional antecedents of sport commitment, such as social support (Berki et al., 2020; Hosseini and Farzan, 2018), mental toughness (Moradi and Zarei, 2018), involvement alternatives (De Francisco et al., 2022; Jeon and Casper, 2016; Weiss and Aloe, 2019), and perceived costs (Weiss, 2020; Weiss et al., 2010).

In addition, the environment appears to be an important factor influencing commitment (Al-Zu’bi et al., 2024; Cao et al., 2024). In sports, this influence is often examined through the lens of the talent development environment (TDE). This is a dynamic system that includes the immediate environment at the micro level, the interactions between these environments, and the cultural context at the macro level (Li et al., 2014). Andronikos et al. (2021) examined the effect of the TDE on sport commitment from a holistic lens. The results showed that long-term development fundamentals, an important component of the TDE (Martindale et al., 2010), have a positive effect on sport commitment.

Additionally, more research has examined the relationship between individual dimensions of the TDE and commitment. For example, Mishra et al. (2016) along with Jang and Seo (2021) argued that the communication environment had a positive effect on commitment. Mazerolle and Dodge (2015) suggested that positive clinical education experiences contributed to enhanced career commitment among students. Furthermore, supportive environment factors, such as role modeling (Winter et al., 2021), coach support (Wekesser et al., 2021), and parental support (Krommidas et al., 2022), have been found to positively affect sport commitment.

However, some researchers hold different views on the effect of various TDE factors on sport commitment. For example, Andronikos et al. (2021) rejected most TDE factors, such as long-term development focus and communication, arguing that there was no significant correlation between them and sport commitment. Some studies have indeed shown that environmental factors such as parenting styles (Vega-Díaz and González-García, 2025) and mental skills training do not significantly affect sport commitment (Arican and Gökyürek, 2022).

The aforementioned studies highlight the theoretical significance of the TDE as an antecedent of sport commitment while reflecting the ongoing academic debate regarding which specific factors within the TDE affect sport commitment. This divergence underscores the need for further research. In particular, research from this perspective remains limited in China (Hong and Li, 2022). Considering the specific characteristics of the Chinese sports system—especially in terms of athlete policy and resource allocation—support tends to be concentrated on few elite athletes who achieve outstanding results. In contrast, the majority of athletes in the “middle” and “lower” tiers often have limited access to such resources (Mengzi, 2023).

Therefore, the primary aim of this study was to examine the effect of various factors of the TDE on the sport commitment of Chinese athletes and whether this effect differed across sport levels. We hypothesized that: (1) TDE factors affected sport commitment; (2) the effect of TDE factors on sport commitment differed between sub-elite and elite athletes.

## Methods

### Participants

The study used random sampling to obtain a sample of 176 athletes from the Hubei Province, China. Their average age was 19 years (SD = 3.16). Of these, 114 were male athletes (64.8%) and 62 female athletes (35.2%); 48 were elite (27.3%) and 128 were sub-elite (72.7%) athletes (Table 1). Participants came from 14 different sport disciplines, including basketball, table tennis, and athletics. To ensure timeliness and relevance of the study, the participating athletes had all regular specialized training experience, with average total duration exceeding five years. Furthermore, a power analysis for the R^2^ test was conducted using Stata 18.0 to estimate the required sample size, with the significance level set at 0.05 and statistical power at 0.80. The R^2^ value for the model was set at 0.23, as determined by the findings of Andronikos et al. (2021). Initially, the model incorporated seven covariates. The required sample size was calculated to be 56, a figure that was substantially lower than the actual sample size of 176 utilized in this study. However, given that the subsample of elite athletes was slightly below this threshold, a recalculation was performed after confirming the final stepwise regression model. When the number of tested covariates was reduced to three, the required sample size was 41, indicating that the elite athlete subsample still met the minimum requirement. Ethical approval for the study was received from the ethics committee of the Hubei University of Technology, Wuhan, China (protocol code: HBUT20240021; approval date: 01 October 2024) (Table 1).

**Table 1:** The study sample information.

Performance Level	Age: M (SD)	Years of Specialized Training: M (SD)	Gender: Number of Participants
Sub-elite athletes	18.6 (2.54)	5.1 (2.32)	Male: 88
			Female: 40
Elite athletes	21.9 (3.39)	6.5 (2.51)	Male: 25
			Female: 23

Note: M = mean; SD = standard deviation

### Measures

#### Demographic Questionnaire

Participants provided demographic information, including age, gender, the sport discipline, the performance level, along with duration of specialized training, to support the continuation of the study.

#### Sport Commitment

This study was based on the research of Scanlan et al. (1993). The questionnaire contained four items to assess athletes’ desire and determination to continue participating in their current sport (e.g., “I am determined to stay in this sport.”). Participants rated their responses on a five-point Likert scale (1 = strongly disagree and 5 = strongly agree). Prior to distribution, the reliability of the questionnaire was tested. The results showed a Cronbach’s α of 0.856, indicating good reliability.

#### Talent Development Environment

Based on the research by Martindale et al. (2010), we used a questionnaire to measure the TDE factors, and it consisted of seven subscales, each designed to assess a specific factor within the TDE. Participants responded using a five-point Likert scale (1 = strongly disagree and 5 = strongly agree). The seven subscales and their details were as follows:

#### Long-Term Development Focus

This subscale included 24 items that assessed the extent to which development opportunities provided to athletes were structured to promote long-term success. It addressed factors such as ongoing opportunities, clear expectations, and the development of attitudes and psychological skills necessary for sustained achievement. The subscale demonstrated excellent reliability, with a Cronbach’s α of 0.972.

#### Quality Preparation

Comprising five items, this subscale evaluated the availability of clear guidance and high-quality practice through training, recovery, and competition experience. It showed good reliability (Cronbach’s α = 0.782).

#### Communication

This subscale contained seven items to assess the effectiveness of formal and informal communication between coaches and athletes. It demonstrated excellent reliability, with a Cronbach’s α of 0.950.

#### Understanding the Athlete

This subscale contained four items to measure the depth of the coach’s understanding of the athlete. It showed good reliability (Cronbach’s α = 0.749).

#### Support Network

This subscale comprised eight items assessing the presence and accessibility of a coherent and broad support network surrounding the athlete. Reliability was good (Cronbach’s α = 0.805).

#### Challenging and Supportive Environment

Containing four items, this subscale evaluated whether there was an appropriately challenging and supportive environment during the athlete’s development. It had acceptable reliability, with a Cronbach’s α of 0.624.

#### Long-Term Development Fundamentals

This subscale included seven items that examined foundational elements critical to further athlete development, such as avoiding early specialization, parental support, and encouraging athletes’ autonomy in decision-making. It demonstrated excellent reliability, with a Cronbach’s α of 0.922.

### Design and Procedures

The dependent variable in this study was sport commitment, and the independent variables were the seven factors comprising the TDE. Data were collected online using the Wenjuanxing platform. The questionnaire included an explanation of the research purpose to ensure that each participant understood the survey content, and some items were reverse-scored. The questionnaire was completed anonymously, and all participants were informed that their participation in the study was voluntary. They could choose to withdraw at any stage.

To test the first hypothesis, Pearson correlation coefficients were used to examine the relationships between the seven factors of the TDE and sport commitment. Subsequently, stepwise regression analysis was conducted using five-fold cross-validation to explore the effect of each TDE factor on sport commitment.

To test the second hypothesis, the sample was divided into two categories based on the athlete’s performance level: sub-elite and elite. The stepwise regression results mentioned earlier were then used to test the subsamples.

### Statistical Analysis

The collected data were imported into Stata 18.0 for subsequent statistical analysis. First, descriptive statistics (i.e., mean and standard deviation) and correlation tests were conducted for each data group (details can be found in Table 2). Long-term development focus, communication, understanding the athlete, support network, and long-term development fundamentals showed a significant positive correlation with sport commitment. Quality preparation and a challenging or a supportive environment were not significantly correlated with sport commitment.

**Table 2:** Descriptive statistics and Pearson correlation coefficients of the research variables.

	M	SD	Min	Max	1	2	3	4	5	6	7	8
1. LTD-Fo	3.89	0.74	1.67	4.96	1	0.072	0.849^***^	0.508^***^	0.499^***^	0.2^***^	0.797^***^	0.709^***^
2. Quality-P	3.07	0.81	1	5		1	0.059	0.327^***^	0.045	0.113	0.067	0.13
3. Com	3.59	0.95	1	5			1	0.544^***^	0.641^***^	0.063	0.789^***^	0.537^***^
4. Und-A	3.37	0.79	1.5	4.5				1	0.458^***^	0.156^**^	0.528^***^	0.382^***^
5. Support-N	3.56	0.53	2	4					1	0.019	0.618^***^	0.199^***^
6. Cha-S	3.61	0.45	2.5	4						1	0.169^**^	0.046
7. LTD-Fu	3.6	0.9	1	5							1	0.594^***^
8. Sport-C	4.03	0.79	1.5	5								1

Note: LTD-Fo = Long-Term Development Focus; Quality-P = Quality Preparation; Com = Communication; Und-A = Understanding the Athlete; Support-N = Support Network; Cha-S = Challenging and Supportive Environment; LTD-Fu = Long-Term Development Fundamentals; Sport-C = Sport Commitment; M = mean; SD = standard deviation; Min = minimum; Max = maximum; ** p < 0.05, *** p < 0.01

Subsequently, due to the high correlations between some TDE factors, we performed multicollinearity diagnostics. The results showed that the variance inflation factor (VIF) values for long-term development focus (VIF = 4.977), communication (VIF = 5.628), and long-term development fundamentals (VIF = 3.83) were greater than 3.0, indicating a certain degree of multicollinearity between these three factors. The remaining VIF values were less than 3.0. Based on these results, stepwise regression was used to examine the effect of each TDE factor on sport commitment (Yu et al., 2014). To ensure the reliability of the study, we also conducted five-fold cross-validation and selected the optimal model from the results (Yu et al., 2014).

Finally, multiple linear regression analysis was conducted on the categorized samples using the selected optimal model to investigate group differences in the effect of the TDE on sport commitment. The significance level for this study was set at 0.05 (Table 2).

## Results

### Stepwise Regression Analysis of the Full Sample

The study used stepwise regression analysis to select the most appropriate variables. To obtain stable and reliable results, we additionally performed five-fold cross-validation and generated five regression models through stepwise regression. The variables included in each model are shown in Table 3. In the table, “1” indicates that the variable was included in the model, while “0” indicates that the variable was not included. Based on related research (Yu et al., 2014), we examined the *F* value and adjusted *R*^2^ of the five models to assess the model fit, as well as the mean absolute percentage error (MAPE) to evaluate the prediction accuracy. The model with the smallest MAPE was selected as the optimal model (Table 3).

**Table 3:** Results of the five-fold cross-validation.

No.	*x*_1_	*x*_2_	*x*_3_	*x*_4_	*x*_5_	*x*_6_	*x*_7_	F	Ad *R*^2^	MAPE	n
1.	1	1	0	0	1	0	0	49.04	0.577	0.112	142
2.	1	0	1	0	1	1	1	61.65	0.636	0.108	140
3.	1	0	1	1	1	1	1	25.03	0.548	0.140	140
4.	1	0	0	0	1	1	1	42.41	0.544	0.102	140
5.	1	0	0	0	1	0	1	38.76	0.588	**0.092**	142

Note: *x*_1_ = Long-Term Development Focus; *x*_2_ = Quality Preparation; *x*_3_ = Communication; *x*_4_ = Understanding the Athlete; *x*_5_ = Support Network; *x*_6_ = Challenging and Supportive; *x*_7_ = Long-Term Development Fundamentals Bold values indicate that the model with the smallest MAPE is selected as the optimal model

In Table 4, model 1 presents the results of the full sample test using the optimal model described earlier. Model 2, based on model 1, includes control variables such as gender, age, the performance level, and the sport discipline. As shown in Table 4, the direction of the coefficients and their significance for each variable were consistent across the two models. In model 1, long-term development focus (*p* < 0.01) and long-term development fundamentals (*p* < 0.01) had a significant positive effect on sport commitment. Conversely, the support network (*p* < 0.01) had a significant negative effect on sport commitment (Table 4).

**Table 4:** Regression coefficients of the optimal model for the full sample.

	Model 1	Model 2
Variable	B (SE B)	B (SE B)
Independent Variables		
1. Long-Term Development Focus	0.115^***^ (0.015)	0.113^***^ (0.015)
2. Support Network	−0.207^***^ (0.048)	−0.199^***^ (0.048)
3. Long-Term Development Fundamentals	0.124^***^ (0.047)	0.124^**^ (0.048)
Control Variables		
1. Age		control
2. Gender		control
3. Performance level		control
4. Sport discipline		control
n	176	176
Ad *R*^2^	0.545	0.554

Note: *** p < 0.01; ** p < 0.05

### Multivariate Linear Regression Analysis for Subsamples

The sample was divided into two groups based on the performance level: sub-elite and elite athletes. The optimal model described earlier was used to examine the group differences in the effect of the TDE on sport commitment. As shown in Table 5, after controlling for gender, age, and the sport discipline, long-term development focus had a significant positive effect on sport commitment for both sub-elite and elite athletes (*p* < 0.01 and *p* < 0.05, respectively). The support network had a significant negative effect on sport commitment for sub-elite athletes (*p* < 0.01). For elite athletes, the effect was not significant. However, the average effect of the elite group was larger in the regression coefficients, which suggests that the lack of significance for this variable was mainly due to the high heterogeneity within the sample. Long-term development fundamentals had a significant positive effect on sport commitment for elite athletes (*p* < 0.05), but no significant effect on sport commitment for sub-elite athletes (Table 5).

**Table 5:** Regression coefficients for subsamples.

	Sub-elite athletes	Elite athletes
Variable	B (SE B)	B (SE B)
Independent Variables		
1. Long-Term Development Focus	0.163^***^ (0.020)	0.064^**^(0.030)
2. Support Network	−0.159^***^ (0.051)	−0.185(0.126)
3. Long-Term Development Fundamentals	−0.040 (0.064)	0.238^**^(0.092)
Control Variables		
1. Age	control	control
2. Gender	control	control
3. Sport discipline	control	control
n	128	48
Ad *R*^2^	0.631	0.449

Note: *** p < 0.01; ** p < 0.05

## Discussion

This study aimed to examine the effect of various TDE factors on the sport commitment of Chinese athletes and whether this effect differed across various sport levels. In the overall data analysis, we used stepwise regression and five-fold cross-validation to select the optimal variables. To ensure the reliability of the study, we included control variables, such as gender, age, the performance level, and the sport discipline, to exclude potential confounding factors (Bernerth and Aguinis, 2016).

Our first hypothesis was partially supported, as only long-term development focus, the support network, and long-term development fundamentals had an effect on sport commitment. Subsequently, the optimal model was used to verify whether the effect of the TDE on sport commitment differed between the two groups. Our second hypothesis was confirmed, showing that the influence of long-term development fundamentals and support network on sport commitment differed between sub-elite and elite athletes.

Long-term development focus emphasizes the long-term and developmental nature of training programs, including continuous opportunities, clear expectations, and the attitudes required to achieve long-term success (Martindale et al., 2010). The results of this study indicate that these factors have a positive effect on sport commitment. This is partly due to the factors that facilitate the formation of approach goals in athletes (Wang et al., 2016). For example, long-term development focus emphasizes allowing athletes to make mistakes and encouraging them to take responsibility for their own development, which increases the likelihood of setting approach goals (Wang et al., 2016). Previous research showed that approach goals presented a significant positive correlation with sport commitment (Simón-Piqueras et al., 2023). When athletes report approach goals, they often experience higher achievement and lower sport devaluation, meaning they are less likely to feel that the sport they are engaged in has lost its value (Isoard-Gautheur et al., 2016). Moreover, coach expectations, emphasized in long-term development focus, serve as another pathway through which the factor positively influences sport commitment. Coach expectations help enhance athletes’ self-efficacy, in turn fostering stronger intrinsic motivation (Weight et al., 2020). According to research, intrinsic motivation has a strong positive correlation with sport commitment (Almagro et al., 2020). Hence, under the current Chinese sports system, long-term development focus has a significant positive effect on sport commitment, and this applies to athletes at any sport level.

The support network emphasizes the importance of coherent, accessible, and extensive support for athletes (Martindale et al., 2010). The results of this study indicate that support networks have a negative effect on sport commitment. This seems to contradict current understanding (Wekesser et al., 2021). A possible explanation is that the specific characteristics of the Chinese sports system have led to this discrepancy. On the one hand, the imposition of support networks may provide encouragement and positive attitudes to athletes, which has been shown to promote higher levels of sport commitment (Wachsmuth et al., 2025). On the other hand, it may also lead to increased pressure (Kneavel, 2021), which tends to negatively affect sport commitment (Dong et al., 2024).

The Chinese sports system emphasizes that athletes should demonstrate high competitive levels as early as possible (Bonal et al., 2020). Accordingly, the imposition of support networks in this system follows the same principle, urging athletes to rapidly improve their competitive abilities to achieve performance results. This often leads to athletes bearing significant psychological pressure, resulting in a decline in sport commitment. It is worth noting that the results of this study indicate that the negative effect of support networks on sport commitment was not statistically significant among elite athletes. However, the average regression coefficient for this group was larger, which indirectly supports the earlier argument that within the Chinese sports system, support networks may exert more pressure than encouragement.

A potential explanation for the non-significant negative effect observed in this group can be the heterogeneity within this sample (Sedgwick, 2015). Nunes et al. (2020) defined heterogeneity as the degree to which a system deviated from a completely uniform state. This suggests that a small subset of elite athletes may have experienced more encouragement and a positive attitude from the support network rather than pressure. The reason for this could be the individualized characteristics of elite athletes (Zentgraf et al., 2024), which result in differences in how they perceive the support network. Further research is needed to investigate the specific mechanisms at play. Although the regression coefficients for the elite sample were larger, the presence of individual differences meant that the regression results for this sample were ultimately non-significant.

Long-term development fundamentals refer to foundational factors essential for an athlete’s continued development, such as avoiding early specialization and encouraging athlete autonomy in decision-making (Martindale et al., 2010). The results of this study showed that long-term development fundamentals had a positive effect on sport commitment, which is consistent with the findings of Andronikos et al. (2021). First, we can explain this from the perspective of avoiding early specialization. Early specialization may lead to negative reactions, such as psychological burnout and physical fatigue in athletes (Deno et al., 2021; Giusti et al., 2020; Rugg et al., 2021), which is often detrimental to improving sport commitment levels (González-Hernández et al., 2021; Gustafsson et al., 2017). Second, encouraging athletes to make their own decisions has also been shown to promote sport commitment (O’Neil and Hodge, 2020). This is because autonomous decision-making enhances athletes’ sense of belonging, which influences their commitment (Sevil-Serrano et al., 2021).

It is worth mentioning that, according to the results of the subgroup analysis, the effect of long-term development fundamentals on sport commitment was only observed in athletes at the elite level. This may be related to the stage of athletes’ development and the particularities of the Chinese sports system. On the one hand, elite athletes have already progressed beyond the early stages of their careers, and the benefits of avoiding early specialization—particularly in mitigating physical and mental fatigue—have begun to emerge, contributing to greater sport commitment. On the other hand, sub-elite athletes are generally still in the early stages of their careers or have only recently moved beyond that phase, with the accumulation of skills and experience remaining their primary focus (Agudo-Ortega et al., 2023; Côté et al., 2007; Rebelo et al., 2025). Therefore, although avoiding early specialization is an important training principle (Agudo-Ortega et al., 2024), its positive effects may not yet be fully evident for this group.

In addition, research by Reverberi et al. (2021) showed that elite athletes possessed better self-regulation abilities than non-elite athletes. This ability allows individuals to adapt to social and physical environments and is considered a key process in psychological function (Vohs and Baumeister, 2016). When elite athletes make decisions autonomously, they monitor their behavior and continuously reflect on the process, making adjustments to their strategies (Ertmer and Newby, 1996; Jordalen et al., 2020; Toering et al., 2009). Non-elite athletes, lacking the corresponding adjustment abilities and experience, may undergo confusion and anxiety when encouraged to make autonomous decisions. This can negatively affect their sport commitment levels.

Furthermore, the current Chinese sports system requires athletes to achieve good competitive results early in their careers (Bonal et al., 2020). However, early diversification in sports may prevent athletes from meeting this requirement within a short time frame (Güllich et al., 2022; Rojas Valverde et al., 2024; Rodriguez-Gomez et al., 2024), leading to a potential loss of opportunities to continue participating in elite sports talent development in China.

This study used data from athletes in the Hubei Province, China, which limits the geographic generalizability of the findings. Additionally, the proportion of elite athletes in the sample was relatively small. Future research should expand the geographic scope and increase the representation of higher-level athletes to strengthen the external validity of the results. Furthermore, TDEs may vary significantly across different sports; for example, popular sports such as table tennis and basketball may provide a more robust development environment than less popular sports such as rock climbing and skateboarding. As a result, the conclusions of this study may vary depending on the sport discipline.

## Conclusions

This study found that certain factors within the TDE influenced athletic commitment. Specifically, the long-term development focus emphasized the progressive nature and development of training programs, and creating a talent development environment that aligned with this focus was shown to be beneficial for increasing athletic commitment, which holds true for athletes at any performance level. In contrast, the application of support networks generally caused athletes to feel pressure rather than encouragement, often leading to a decline in athletic commitment. However, among elite athletes, this pattern appeared to be moderated by individual differences. The long-term development fundamentals emphasized the importance of avoiding early specialization and encouraging athletes to make autonomous decisions. These practices would help improve athletes’ sense of belonging, reduce physical and mental fatigue, and increase athletic commitment. This effect was more significant for elite athletes.

Therefore, Chinese coaches and relevant professionals should strengthen the creation of TDEs that meet the requirements of long-term development focus, such as allowing athletes to make mistakes and encouraging them to take responsibility for their development. Chinese sports institutions should improve their training philosophies, emphasize athletes’ long-term success and sustainable development, avoid overemphasizing short-term success, and reduce their psychological pressure. Moreover, relevant Chinese institutions should work to improve the elite sports talent development system to align more closely with the principles of long-term development fundamentals, ensuring that athletes are not excluded from the development pathway simply because they do not achieve significant results early in their careers.

Future research should aim to systematically replicate the current study design, incorporate a broader geographic sample, and include a larger proportion of high-level athletes to establish the external validity of the current findings.
